# Flexible structure learning under uncertainty

**DOI:** 10.3389/fnins.2023.1195388

**Published:** 2023-08-03

**Authors:** Rui Wang, Vael Gates, Yuan Shen, Peter Tino, Zoe Kourtzi

**Affiliations:** ^1^State Key Laboratory of Brain and Cognitive Science, CAS Center for Excellence in Brain Science and Intelligence Technology, Institute of Psychology, Chinese Academy of Sciences, Beijing, China; ^2^Department of Psychology, University of Chinese Academy of Sciences, Beijing, China; ^3^Institute for Human-Centered AI, Stanford University, Stanford, CA, United States; ^4^School of Science and Technology, Nottingham Trent University, Nottingham, United Kingdom; ^5^School of Computer Science, University of Birmingham, Birmingham, United Kingdom; ^6^Department of Psychology, University of Cambridge, Cambridge, United Kingdom

**Keywords:** structure learning, uncertainty, perceptual decisions, decision strategy, vision

## Abstract

Experience is known to facilitate our ability to interpret sequences of events and make predictions about the future by extracting temporal regularities in our environments. Here, we ask whether uncertainty in dynamic environments affects our ability to learn predictive structures. We exposed participants to sequences of symbols determined by first-order Markov models and asked them to indicate which symbol they expected to follow each sequence. We introduced uncertainty in this prediction task by manipulating the: (a) probability of symbol co-occurrence, (b) stimulus presentation rate. Further, we manipulated feedback, as it is known to play a key role in resolving uncertainty. Our results demonstrate that increasing the similarity in the probabilities of symbol co-occurrence impaired performance on the prediction task. In contrast, increasing uncertainty in stimulus presentation rate by introducing temporal jitter resulted in participants adopting a strategy closer to probability maximization than matching and improving in the prediction tasks. Next, we show that feedback plays a key role in learning predictive statistics. Trial-by-trial feedback yielded stronger improvement than block feedback or no feedback; that is, participants adopted a strategy closer to probability maximization and showed stronger improvement when trained with trial-by-trial feedback. Further, correlating individual strategy with learning performance showed better performance in structure learning for observers who adopted a strategy closer to maximization. Our results indicate that executive cognitive functions (i.e., selective attention) may account for this individual variability in strategy and structure learning ability. Taken together, our results provide evidence for flexible structure learning; individuals adapt their decision strategy closer to probability maximization, reducing uncertainty in temporal sequences and improving their ability to learn predictive statistics in variable environments.

## Introduction

Successful everyday interactions entail that we identify spatiotemporal regularities (i.e., patterns that repeat frequently) in our cluttered and dynamic environments and exploit them to predict future events. Learning and experience are known to facilitate our ability to extract the environment’s statistics (Perruchet and Pacton, 2006; Aslin and Newport, 2012). For example, humans become sensitive to stimuli (shapes, tones or syllables) that co-occur following a spatial or temporal pattern through repetitive exposure (Saffran et al., 1996a,^1999^; Chun, 2000; Fiser and Aslin, 2002; Turk-Browne et al., 2005).

Our recent work demonstrates that individuals extract the statistics that govern the temporal structure of events and exploit them to make predictions about future events (Wang et al., 2017a). Further, we show that this learning of predictive structures relates to the decision strategy of individuals. In particular, previous work has highlighted the role of strategies in probabilistic learning and decision making (Shanks et al., 2002; Erev and Barron, 2005; Acerbi et al., 2014; Murray et al., 2015; Schulze et al., 2015). Humans and animals are known to engage in probability matching (match their choices probabilistically according to the underlying input statistics) or probability maximization (maximize their success by selecting the most probable outcomes) when making choices. Previous work on decision making and learning has suggested that experience shapes the selection of decision strategies (Rieskamp and Otto, 2006; Fulvio et al., 2014). For example, humans adopt different decision strategies to maximize performance and reduce uncertainty given the demands of the training task. Yet, the factors that determine individual decision strategies and influence structure learning ability remain largely unknown.

Previous work provides evidence for the role of uncertainty in perceptual decision making (Bach and Dolan, 2012). Uncertainty may arise from sensory input and/or outcome. In particular, noisy sensory signals (Dosher and Lu, 1998; Dayan and Daw, 2008; Hasson, 2017; Daikoku, 2018) or increased stochasticity in temporal sequences (Rolke and Hofmann, 2007; Nobre and van Ede, 2018) impact the difficulty of perceptual tasks. Further, informative feedback is known to play a key role in resolving uncertainty and facilitating perceptual decisions (Kluger and DeNisi, 1996; Dayan and Abbott, 2001; Petrov et al., 2005).

Here, we test whether sensory uncertainty and feedback affect decision strategy and structure learning in the context of a sequence prediction task. In particular, we trained participants with temporal sequences comprising unfamiliar symbols and determined by first-order Markov models. Participants were exposed to these context-based statistics (i.e., symbol probability is contingent on previous symbols) and they were asked to judge whether a test symbol that followed the sequence presentation matched the expected symbol based on the preceding sequence. This sequence prediction task allows us to track participant responses over time and interrogate the decision strategy that individuals adopted during learning. We introduced uncertainty in the task by manipulating: (a) the probability of symbol co-occurrence, (b) the stimulus presentation rate (i.e., introducing jitter in stimulus presentation time). Further, we tested the effect of feedback on decision strategy and structure learning. We reasoned that during training individuals will adapt their decision strategies and performance in the sequence prediction task. Our results demonstrate that: (1) increasing the similarity in the probabilities with which symbol contingencies appear in the sequence impaired performance on the prediction task; (2) increasing uncertainty in stimulus presentation rate by temporal jittering facilitated probability maximization and performance; (3) trial-by-trial feedback enhanced performance compared to block feedback or no feedback and facilitated probability maximization, while uncorrelated feedback resulted in limited improvement. Correlating individual strategies with post-training performance showed that observers that adopted a strategy closer to maximization showed better learning performance. Finally, we show that attentional skill may account for individual differences in decision strategy and structure learning ability.

## Materials and methods

### Observers

A total of 105 observers (40 males and 65 females, mean age = 22.1 ± 0.3 years) participated in this study and they were randomly allocated into different experimental groups. All observers were naive to the aim of the study, had normal or corrected-to-normal vision and gave written informed consent. This study was approved by the University of Cambridge Ethics Committee and the institutional review board of the Institute of Psychology, Chinese Academy of Sciences.

### Stimuli

**FIGURE 1:**
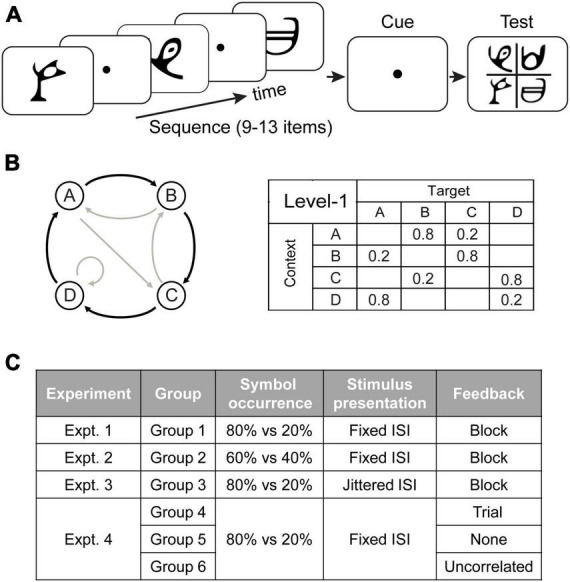
Trial and experimental design. **(A)** A total of 9 to 13 symbols were presented one at a time in a continuous stream followed by a cue and the test display. **(B)** Sequence design. For the first-order Markov model (Level1), a diagram indicates states (circles) and transitional probabilities (black arrow: high probability, e.g., 80%; gray arrow: low probability, e.g., 20%). Transitional probabilities are shown in a four-by-four conditional probability matrix, with rows indicating temporal contexts and columns indicating the corresponding targets. **(C)** Experimental design. In Experiment 1 (Group 1), a sequence of symbols with context-conditional probability of 80% vs. 20% were presented one after another with a fixed ISI. Block feedback (i.e., score in the form of performance index) was provided during training. In Experiment 2 (Group 2), the symbol transitional probability was modified to 60% vs. 40%. In Experiment 3 (Group 3), visual stimuli appeared in a stream separated by jittered ISI. In Experiment 4, we manipulated feedback: Group 4 was trained with trial-by-trial feedback based on the symbol expected by the pre-defined sequences, Group 5 was not provided with any feedback, and Group 6 was trained with random feedback which was uncorrelated to the participants’ responses.

Stimuli comprised 4 symbols chosen from Sabaean alphabet and Ndjuká syllabary (Figure 1A). These symbols were highly discriminable from each other and were unfamiliar to the observers. Each symbol was presented at 6.5*^o^* of visual angle in black on mid-gray background. Experiments were controlled using Matlab and the Psychophysics toolbox 3 (Brainard, 1997; Pelli, 1997). Stimuli were presented on a 21-inch CRT monitor (1,024 × 768 pixel, 100 Hz frame rate) at a distance of 60 cm.

### Sequence design

We employed first-order Markov model (i.e., level-1) to generate probabilistic sequences (Wang et al., 2017a). The level-1 Markov model produces a sequence of symbols, where the symbol at time *i* is determined probabilistically by the immediately preceding symbol. We refer to the symbol presented at time *i*, *s*(*i*), as the *target* and to the previous symbol *s*(*i*-1) as the *context*:


P⁢(s⁢(i)|s⁢(i-1),s⁢(i-2),…,s⁢(1))=P⁢(s⁢(i)|s⁢(i-1)).

At each time point in the sequence, the symbol that follows a given context is determined probabilistically. The underlying Markov model can be represented through the associated context-conditional target probabilities. We used 4 symbols that we refer to as stimuli A, B, C, and D. The correspondence between stimuli and symbols was counterbalanced across participants. Specifically, for level-1, the target depended on the item that immediately preceded it. Given a context (the last seen symbol), only one of two targets could follow (Figure 1B): one had a high probability of being presented (e.g., 80% of occurrence) and the other a low probability (e.g., 20% of occurrence). For example, when Symbol A was presented, only symbols B or C were allowed to follow, and B had a higher probability of occurrence than C.

### Experimental design

We tested six groups of participants (Figure 1C). Experiment 1 (Group 1: *N* = 18) aimed to (a) replicate our previous findings showing that exposure to temporal sequences facilitates observers’ ability to extract temporal structure for making predictions (Wang et al., 2017a), and (b) test whether learning is maintained over time. Specifically, for each trial, symbols (with probabilities of context-target contingencies at 80% vs. 20%) were presented successively with a fixed interstimulus interval (ISI). Feedback was given at the end of each training block. In Experiments 2–4, we manipulated: (1) context-conditional probability of symbol occurrence in sequences; (2) stimulus presentation rate; (3) feedback (for more details see “Training sessions”). Stimuli and procedures in Experiment 2–4 were identical to Experiment 1 (a baseline experiment) apart from the above design manipulations. In Experiment 2 (Group 2: *N* = 18), we modified the symbol transitional probability from 80% vs. 20% to 60% vs. 40%. In Experiment 3 (Group 3: *N* = 18), visual stimuli appeared in a stream separated by jittered ISI. In Experiment 4, we examined the role of feedback in learning predictive structures: In contrast to participants in Group 1 who were trained with block feedback, participants in Group 4 (*N* = 18) were trained with trial-by-trial feedback, participants in Group 5 (*N* = 15) were trained without any feedback, and participant in Group 6 (*N* = 18) were trained with uncorrelated feedback.

All participants underwent six sessions: one session involved testing on cognitive tasks (i.e., working memory and selective attention), the remaining five sessions involved testing and training on the sequence prediction task using first-order Markov sequences. Before and after training (pre- and post-training sessions), participants were tested with structured sequences and random sequences (i.e., all four symbols were presented with equal probability 25% in a random order). To investigate whether the learning effect was maintained over time, ten observers in Group 1 were re-tested 4 weeks after training.

Training sessions: Training comprised 23 blocks of structured sequences (60 trials per block) that were conducted on four consecutive days. For each trial (Figure 1A), a sequence of 9–13 stimuli appeared in the center of the screen, one at a time in a continuous stream, for 100 ms each followed by a central white fixation dot (i.e., ISI) for 400 ms on average. The ISI was fixed at 400 ms, except for Group 3 in which the ISI was jittered; that is the ISI in a given trial was chosen randomly from a uniform distribution of values ranging between 100 and 700 ms and binned in temporal windows of 20 ms (i.e., 100, 120, 140 ms etc.). The end of each trial was indicated by a red-dot cue that was presented for 400 ms. Following this, all four symbols appeared in the center (2 × 2 grid) of the screen. Observers were asked to indicate which symbol they expected to appear following the preceding sequence by pressing a key corresponding to the location of the predicted symbol. If no response was made within 2 s, a null response was recorded and equal probabilities for each symbol (0.25) were registered for this trial for further behavioral analyses. The proportion of trials without responses was 0.9%. Following the observer’s response, a circle appeared on the selected stimulus for 300 ms to highlight the observer’s choice. For Group 4 and Group 6, trial-by-trial feedback was provided by coloring this circle (green vs. red signified “correct” vs. “incorrect” responses, respectively). For Group 4 feedback was based on the symbol determined by the pre-defined sequences using Markov models (Group 4: trial feedback). For example, given a certain context A, symbol B follows with 80% probability. If the participants select B consistently, they will be “correct” 80% of the time and “incorrect” 20% of the time. For Group 6 feedback was uncorrelated to observers’ responses (Group 6: uncorrelated feedback). For other groups (Group 1–3, 5), the color of the circle was always white, simply indicating the observer’s choice rather than providing feedback. Observers were given feedback (i.e., score in the form of performance index (PI), see “Behavioral analysis”) at the end of each block for Group 1–3 (block feedback). Neither block feedback nor informative trial feedback was provided for Group 5 (no feedback).

Test sessions: To compare performance before and after training, the pre- and post-training sessions included three blocks, that is, two blocks of structured sequences interleaved with one block of random sequences (i.e., all four symbols were presented with equal probability 25% in a random order). Participants were trained with structured sequences and tested with both structured and random sequences to ensure that training was specific to the trained sequences. Each block comprised 40 trials, during which participants performed the same sequence prediction task as in the training sessions. The stimuli and procedure were identical to the training sessions but no feedback was given during test sessions.

### Cognitive testing

#### Memory: visual short-term memory

The working memory task was designed based on the sequential working memory task by Luck and Vogel (1997). Colored dots were displayed on a gray background for 500 ms, followed by an inter-stimulus interval of 1,000 ms. Then the dot display re-appeared with one of the dots highlighted by a white square. Participants reported whether the highlighted dot had remained the same color on the second presentation. An initial display of two dots was used. We manipulated the number of dots in the display using a two-down one-up staircase, resulting in 70.7% performance. Working memory thresholds (i.e., number of dots in the display) were calculated by averaging the last two-third reversals in each staircase. For each trial, each dot was randomly assigned a color, and one dot was randomly chosen as the target. Each dot had a radius of 0.44*^o^* in visual angle and dots were displayed in random locations within a 10 × 10 grid (jittered ± 0.36*^o^*). Each run consisted of 10 staircase reversals, and participants completed 3 runs, after which we computed the average threshold as their working memory score. In this task, a higher score (greater number of items in display) denotes better performance.

#### Attention: useful field of view

We used the Useful Field of View task to assess selective attention (Edwards et al., 2005, 2006). This task was performed using the UFOV^®^ testing software (Visual Awareness Inc.) and the refresh rate of the monitor was set to 60 Hz as required. Each trial started with a fixation bounding box (1-s duration), followed by the test stimuli (variable duration between 16.7 and 500 ms), a white noise visual mask to control for after images (1-s duration) and the response screen (displayed until a response was made). The central stimulus (a silhouette of 1.9*^o^* × 1.4*^o^* of a car or a truck) was presented on a black background inside a white bounding box, with a simultaneously presented peripheral stimulus (1.9*^o^* × 1.4*^o^* silhouette of a vehicle) which was fixed at 10*^o^* retinal eccentricity from the central stimulus at one of the eight radial locations. The target stimuli were embedded in the context of distractors (47 triangles of the same size and luminance as the targets). Participants were asked to ignore the triangles and point out whether the central stimulus comprised a car or a truck, as well as the location of the peripheral target. Using a double-staircase method, the duration of the display within each task varied between 16.7 and 500 ms. This allowed us to establish the minimal display duration at which the participant could correctly perform the tests 75% of the time. Participants completed three runs, after which we computed the average threshold as their selective attention score. Thus, a lower score (shorter display duration) indicates better performance in this task.

### Behavioral analysis

#### Performance index (PI)

We assessed participant responses in a probabilistic manner, following our previous work (Wang et al., 2017a). We computed a performance index per context that quantifies the minimum overlap (min: minimum) between the distribution of participant responses (P_resp_) and the distribution of presented targets (P_pres_) estimated across 60 trials per block by:


$$\mathrm{PI}\left(\mathrm{context}\right)=\sum_{t\,a\,r\,g\,e\,t}min(P_{r\,e\,s\,p}\left(target|context\right),$$

$$P_{p\,r\,e\,s}\left(target|context\right))$$

The overall performance index is then computed as the average of the performance indices across contexts, PI(context), weighted by the corresponding context probabilities P(context):


$$\text{PI}=\sum_{c\,o\,n\,t\,e\,x\,t}P\,I\,\left(c\,o\,n\,t\,e\,x\,t\right)\cdot P\,\left(c\,o\,n\,t\,e\,x\,t\right)$$

To compare across different conditions, we defined a normalized PI measure that quantifies participant performance relative to random guessing. We computed a random guess baseline; i.e., performance index PI_rand_ that reflects participant responses to targets with equal probability for each target for a given context for level-1 (PI_rand_ = 0.45 for probability of 80% vs. 20%, PI_rand_ = 0.50 for probability of 60% vs. 40%). To correct for differences in random-guess baselines, we subtracted the random guess baseline from the performance index (PI_normalized_ = PI–PI_rand_). PI improvement was the difference in normalized PI between pre- and post-training sessions. We used 10% above chance after training (i.e., PI_normalized_ ≥ 10% at the post-training session) as criterion for learning. Participants who did not meet this criterion were identified as weak learners.

#### Strategy choice and strategy index

Following our previous work (Wang et al., 2017a), we quantified each participant’s strategy, by comparing individual participant response distributions (response-based model) to two baseline models: (i) probability matching, where probabilistic distributions are derived from the Markov models that generated the presented sequences (Model-matching) and (ii) a probability maximization model, where only the single most likely outcome is allowed for each context (Model-maximization). We used Kullback–Leiber (KL) divergence to compare the response distribution to each of these two models. KL is defined as follows:


$$K\,L=\sum_{c\,o\,n\,t\,e\,x\,t}M\,\left(c\,o\,n\,t\,e\,x\,t\right)\,\sum_{t\,a\,r\,g\,e\,t}M\,\left(t\,a\,r\,g\,e\,t|c\,o\,n\,t\,e\,x\,t\right)$$

$$l\,o\,g\,\left(\frac{M\,\left(t\,a\,r\,g\,e\,t|c\,o\,n\,t\,e\,x\,t\right)}{R\,\left(t\,a\,r\,g\,e\,t\right)|c\,o\,n\,t\,e\,x\,t}\right)$$

for level-1 model where R() and M() denote the conditional probability distribution derived from the human responses and the models (i.e., probability matching or maximization), respectively, across all the conditions.

We quantified the difference between the KL divergence from the response-based model to Model-matching and the KL divergence from the response-based model to Model-maximization. We refer to this quantity as strategy choice indicated by ΔKL(Model-maximization, Model-matching). We computed strategy choice per training block, resulting in a strategy curve across training for each individual participant. We then derived an individual strategy index by calculating the integral of each participant’s strategy curve and subtracting it from the integral of the exact matching curve, as defined by Model-matching across training. We defined the integral curve difference (ICD) between individual strategy and exact matching as the individual strategy index. Therefore, strategy index is a continuous measure that captures the strategy that individuals adopt over time on a continuous scale between matching and maximization. Zero strategy index indicates that the participant response distribution matches the probability distribution of the presented sequence. Participant performance deviating from the matching model may result in a positive or negative strategy index. Overestimating the probability of the most probable context-target contingency in the sequence results in a positive strategy index, indicating that the participant’s strategy ranges between matching and maximization. In contrast, underestimating the probability of the most probable context-target contingency in the sequence results in a negative strategy index, indicating that the participant’s strategy ranges between matching and a random model of response (that is, participants choose all context-target contingencies with equal probability). Thus, we interpret strategy index values close to zero as strategy closer to matching, and higher positive values as strategy deviating from matching toward maximization.

Although both performance index and strategy index provide a quantitative evaluation of the difference between the probability distributions of context-target contingencies corresponding to the generative sequence models and the participant responses, they serve different purposes. In particular, PI employs the minimum overlap between the two distributions, as an indication of how close model and response distributions match. Strategy index indicates the difference between participant responses and a reference model (i.e., matching or maximization model), using a stricter information-theoretic evaluation of the distributional difference, as higher nuance is needed for evaluating whether the participant responses follow either the generative model, the maximization approach, or uniformly random guessing.

### Statistical analysis

We conducted repeated measures ANOVA and *t*-tests in IBM SPSS. All statistical tests were two tailed. We also conducted Bayesian statistics in JASP (JASP Team, 2023, Version 0.17.2) with default Cauchy prior (*r* scale = 0.707). The Bayes factor (BF_10_) quantifies the strength of evidence in favor of the data supporting the alternative rather than null hypothesis: BF_10_ < 1 provides evidence favoring the null hypothesis with BF_10_ between 1/10 and 1/3 providing substantial evidence for the null hypothesis (Kass and Raftery, 1995; Wagenmakers et al., 2011); while BF_10_ > 1 provides evidence favoring the alternative hypothesis.

## Results

### Experiment 1: learning temporal statistics with block feedback

**FIGURE 2:**
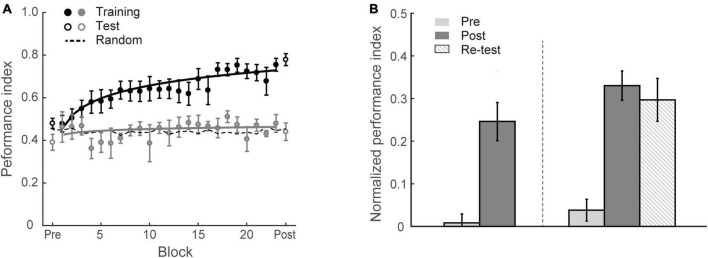
Experiment 1. **(A)** Behavioral performance. Performance index is shown across training (solid circles) blocks, pre-training test and post-training test (open circles). Data are fitted separately for participants who improved during training (learners, black symbols, *N* = 13) and those who did not improve (weaker learners, gray symbols, *N* = 5). Random guess baseline is indicated by dotted lines. **(B)** Normalized PI for test sessions. Data are shown for all participants in Group 1 (*N* = 18, left panel) and those who completed re-test session (*N* = 10, right panel). Performance is shown before (gray bars), immediately after (black bars) and 4 weeks after training (dotted bars). Error bars indicate standard error of the mean.

To test whether individuals adapt to the environment’s statistics, we trained eighteen participants (Group 1) on multiple training blocks over four sessions, during which they were presented with structured sequences of symbols that were determined by the first-order Markov model (i.e., context length of 1, the occurrence of a particular symbol is conditionally dependent on the immediately preceding symbol) and were asked to perform a prediction task; that is, participants indicated the symbol they expected to appear following the preceding sequence. During the training phase, the visual stimuli were presented one after another at a fixed rate of 2 Hz. Participants were given block feedback; that is, the Performance Index (PI) score (indicating how closely the probability distribution of participant responses matches the probability distribution of the presented symbols) was shown to the participants at the end of each block (i.e., 60 trials). For example, random guess with equal probability for each target at a given context (80% vs. 20% of 1st order Markov model) results in PI of approximate 0.45; PI of 1 indicates perfect matching between probability distributions for participant’s responses and presented sequences. Higher PI indicates better performance. To quantify the learning effect, we compared the normalized PI (i.e., after subtracting performance based on random guessing) before and after training. A repeated measures ANOVA showed a significant session effect [*F*(1,17) = 36.72, *p* < 0.001], indicating that repeated exposure facilitates learning of structured sequences (Figure 2). Specifically, most observers (13/18) improved in the prediction task; only five participants showed performance less than 10% above random guessing (i.e., weak learners) after training. The mean PI improvement of Group 1 was 23.5 ± 3.9%. The learning curves in Figure 2A indicate that performance improves throughout training. These results corroborated our previous findings (Wang et al., 2017a) showing that participants succeed in extracting regularities and making predictions about upcoming events.

To examine whether the learning effect we observed was maintained over time, ten participants were called back for an additional test session 4 weeks after training (spaced by 27.2 ± 4.8 days). Performance in this test session was significantly higher than the pre-training test [*F*(1,9) = 38.54, *p* < 0.001] (Figure 2B). Mean PI improvement immediately after and 4 weeks after training was 29.2 ± 3.4% and 25.9 ± 4.2%, respectively and did not differ significantly between these post-tests [*t*(9) = 1.06, *p* = 0.319, BF_10_ = 0.486], suggesting that the training-dependent improvement we observed was sustained for a prolonged time.

### Experiment 2: manipulating context-conditional probability of symbol occurrence

In this experiment we asked whether increasing uncertainty during the training by manipulating the context-conditional probability of symbol occurrence affects learning in the context of the prediction task. In particular we changed the symbol transitional probability from 80% vs. 20% (Experiment 1) to 60% vs. 40%, while keeping the context and targets identical to the model used in Experiment 1. We hypothesized that decreasing the discriminability of contingency probabilities would impair learning. We trained a new group of eighteen observers (Group 2) on the prediction task using the less discriminable contingency probabilities (60% vs. 40%).

**FIGURE 3:**
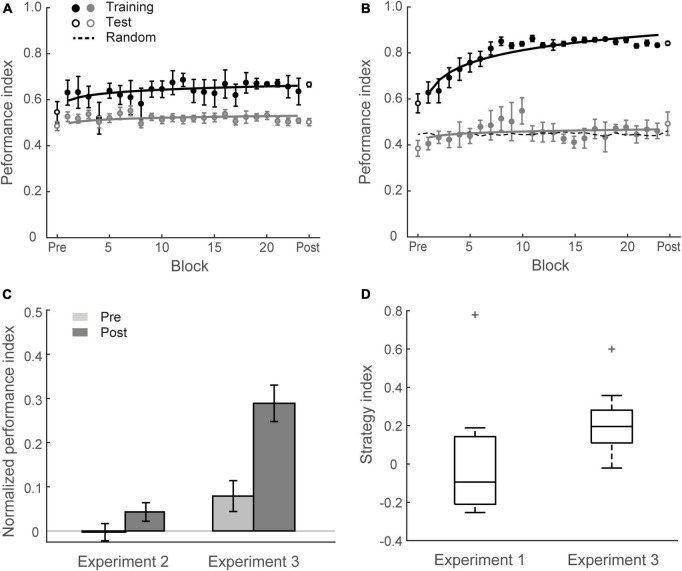
Experiment 2 and Experiment 3. **(A)** Mean Performance index across test and training blocks for Group 2 (Experiment 2). Data are fitted separately for participants who improved during training (learners, black symbols, *N* = 5) and those who did not improve (weak learners, gray symbols, *N* = 13). **(B)** Mean Performance index across test and training blocks for Group 3 (Experiment 3). Data are fitted separately for participants who improved during training (black symbols, *N* = 13) and those who did not improve (gray symbols, *N* = 5). **(C)** Normalized PI pre- and post-training for Group 2 and Group 3. Data are shown for all participants. Error bars indicate standard error of the mean. **(D)** Box plots of strategy index show individual variability for learners in Group 3 and Group 1 (Experiment 1). The upper and lower error bars display the minimum and maximum data values, and the central boxes represent the interquartile range (25th–75th percentiles). The thick line in the central boxes represents the median. Crosses denote outliers.

**FIGURE 4:**
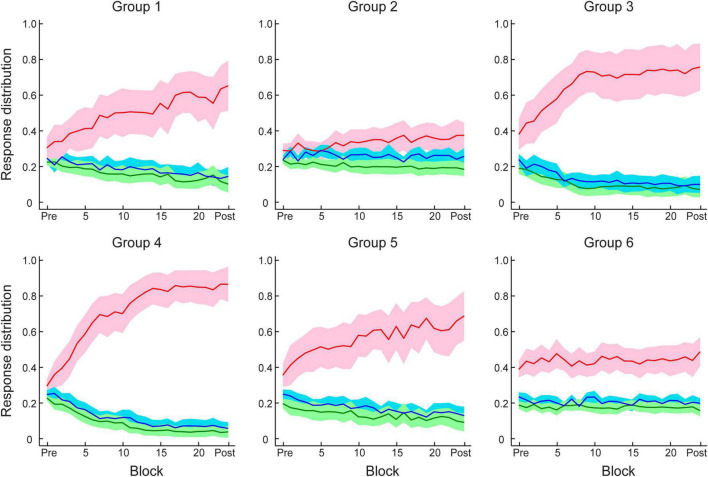
Participant response distributions for conditional probabilities of context–target contingencies across test and training blocks in Experiment 1–4. Red and blue lines indicate the conditional probabilities derived from participant responses of the frequent (e.g., 80%) and infrequent (e.g., 20%) symbols, respectively, for the given contexts. Green lines indicate the averaged probability of responding to one of the two symbols that were not allowed as next symbols for the given contexts. Solid lines indicate mean across participants and shading indicates 95% CI.

Figures 3A, C show that this manipulation resulted in low PI improvement (4.6 ± 2.2%). A two-way mixed ANOVA comparing performance before and after training between Group 1 (Experiment 1) and Group 2 (Experiment 2) showed a significant interaction of session and group [*F*(1,34) = 18.12, *p* < 0.001]. There was no significant difference between groups for the pre-training performance [*F*(1,34) = 0.11, *p* = 0.738]. In contrast, performance after training for Group 1 was significantly higher than Group 2 [*F*(1,34) = 16.58, *p* < 0.001]. These results suggest that probability of context-target contingencies affects learning of temporal statistics; that is, making the probabilities of symbol co-occurrence less discriminable compromises performance and learning in the prediction task. Figure 4 demonstrates participants’ ability to extract context-target contingencies over time across groups. Tracking response probability distributions over training shows that participants did not simply underestimate (e.g., 50% vs. 50%) or overestimate (e.g., 70% vs. 30%) the probabilities of the contingencies. In contrast, they responded close to random-guess (i.e., nearly equal probability for each symbol in a given context), suggesting that less discriminable probabilities of symbol co-occurrence (i.e., Experiment 2) make it difficult to extract the behaviorally relevant statistics.

### Experiment 3: manipulating uncertainty in stimulus presentation rate by temporal jitter

Previous studies have shown that rhythmic stimulation that induces strong temporal expectation facilitates processing of events (Jones et al., 2002; Schroeder and Lakatos, 2009; Rohenkohl et al., 2012). Here, we tested whether disrupting rhythmic stimulation by introducing temporal jitter disrupts learning in the prediction task. In particular, we varied the ISI between successive stimuli in a trial. That is, in Group 1, the stimuli were presented at a fixed rate of 2 Hz and the ISI was fixed at 400 ms. In contrast, in Experiment 3 (*N* = 18, Group 3) the ISI was jittered, ranging from 100 to 700 ms. We reasoned that jittering the ISI would prevent temporal expectation and may impair learning of temporal statistics.

In contrast to this prediction, our results show that learning was maintained when participants exposed to sequences of symbols at an arrhythmic presentation rate (i.e., there was no significant difference between Group 1 and Group 3). Specifically, training resulted in a significant improvement for most participants in Group 3 (13/18) except five participants who showed little improvement (i.e., performance after training less than 10% above random guessing) (Figure 3B). The mean PI improvement of Group 3 was 21.0 ± 3.5%. A two-way mixed ANOVA comparing across Experiment 1 and Experiment 3 with Session (Pre vs. Post) and Group (Group 1 vs. Group 3) showed a significant main effect of session [*F*(1,34) = 72.81, *p* < 0.001], consistent with enhanced performance after training. There was no significant main effect of group [*F*(1,34) = 1.79, *p* = 0.190] nor interaction between session and group [*F*(1,34) = 0.22, *p* = 0.639], indicating similar improvement across groups despite temporal jitter in Group 3 [*t*(34) = 0.47, *p* = 0.639, BF_10_ = 0.351].

### Decision strategies for learning temporal statistics

We next asked whether increasing temporal uncertainty by manipulating the stimulus presentation rate affects participant decision strategies when making predictions. Previous work on probabilistic learning and decision making has proposed that individuals adopt decision strategies ranging from matching to maximization when making probabilistic choices (Shanks et al., 2002; Erev and Barron, 2005; Acerbi et al., 2014; Murray et al., 2015; Schulze et al., 2015). We have previously shown that in the context of our prediction task, participants are exposed to stochastic sequences and use these strategies when learning the probabilities of different outcomes (Wang et al., 2017a). Modeling the participants’ responses allows us to quantify their decision strategy. Specifically, participants may adopt (1) probability matching (that is, match their choices to the relative probabilities of the context-target contingencies presented in the sequences); or (2) deviate from matching toward maximization (that is, choose the most likely outcome in a given context). To quantify these strategies, we computed a strategy index that indicates participant’s preference (on a continuous scale) for responding using probability matching versus maximization. Figure 3D illustrates variability of strategy index for learners from Group 1 and Group 3. The strategy index for Group 1 was not significantly different from matching [that is, zero strategy index; *t*(12) = 1.32; *p* = 0.213, BF_10_ = 0.567], while the strategy index for Group 3 was significantly higher than zero [*t*(12) = 8.93; *p* < 0.001]. Comparing individual strategy across groups showed significantly higher strategy index for Group 3 than Group 1 [*t*(24) = 3.14, *p* = 0.004], suggesting that disrupting rhythmic stimulation by temporal jitter results to a decision strategy closer to maximization.

Correlating learning performance (i.e., Normalized PI after training) with strategy index showed a significant positive relationship (*r* = 0.754, *p* < 0.001 for Group 1 and Group 3, *N* = 36), suggesting that maximization strategy relates to improved performance in the prediction task. This relationship may explain the surprising result we observed for Group 3; that is, learning was maintained when temporal jitter was introduced. Adopting a strategy closer to maximization may facilitate learning when uncertainty in stimulus presentation rate is increased due to temporal jitter.

### Experiment 4: manipulating feedback

**FIGURE 5:**
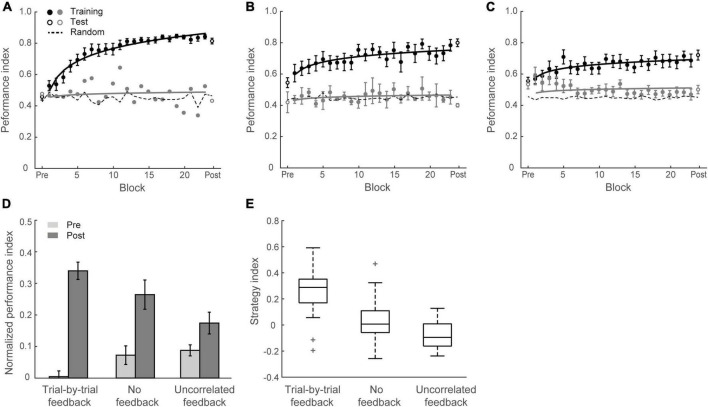
Experiment 4. **(A)** Mean Performance index across blocks for Group 4 (trial-by-trial feedback). Data are fitted separately for participants who improved during training (learners, black symbols, *N* = 17) and one participant who did not improve (weak learners, gray symbols). **(B)** Mean Performance index across blocks for Group 5 (no feedback). Data are fitted separately for participants who improved during training (*N* = 12) and those who did not improve (*N* = 3). **(C)** Mean Performance index across blocks for Group 6 (uncorrelated feedback). Data are fitted separately for participants who improved during training (*N* = 10) and those who did not improve (*N* = 8). **(D)** Normalized PI for test sessions in Groups 4, 5, and 6. Data are shown for all participants. Error bars indicate standard error of the mean. **(E)** Box plots of strategy index show individual variability per group.

Theoretical work has suggested that supervised, error-correcting learning mechanisms rely on external feedback (Dayan and Abbott, 2001). To understand the role of feedback in learning temporal statistics, we trained three additional groups of participants with (a) trial-by-trial feedback based on the symbol expected by the pre-defined sequences (Group 4, Figure 5A); (b) no feedback (Group 5, Figure 5B); (c) uncorrelated feedback, that is, random trial-by-trial feedback that was uncorrelated to the observers’ responses (Group 6, Figure 5C).

Figure 5D shows mean performances before and after training per group. A two-way mixed ANOVA showed a significant interaction of session and group [*F*(2,48) = 12.69, *p* < 0.001], suggesting that performance improvement differed across groups. PI improvement for Group 4, Group 5, and Group 6 was 33.2 ± 3.6%, 19.0 ± 3.9%, and 8.6 ± 3.3%, respectively. Specifically, trial-by-trial feedback (Group 4) resulted in most participants (17/18 learners) showing improvement in the task that was on average higher than the improvement observed for the other groups [Group 4 vs. Group 5: *t*(31) = 2.70, *p* = 0.011; Group 4 vs. Group 6: *t*(34) = 5.04, *p* < 0.001]. Most participants improved in the task even without feedback (12/15 learners) and there was no significant difference in performance between block feedback and no feedback [Group 1 vs. Group 5: *t*(31) = 0.81, *p* = 0.427, BF_10_ = 0.428]. However, providing uncorrelated feedback resulted in limited improvement [i.e., two thirds participants showed < 10% improvement; Group 5 vs. Group 6: *t*(31) = 2.06, *p* = 0.047], and nearly half participants (8/18) showed performance less than 10% above random guessing in the prediction task after training.

We then compared participant decision strategies across groups to test whether feedback modulates decision strategy (Figures 4, 5E). A one-way ANOVA on strategy index showed a significant effect of group [*F*(2,48) = 15.70, *p* < 0.001]. For participants who trained with trial-by-trial feedback (Group 4), the strategy index was significantly higher than zero [*t*(17) = 5.23, *p* < 0.001] and higher than the strategy index for groups that trained with no feedback or uncorrelated feedback [Group 4 vs. Group 5: *t*(31) = 2.94, *p* = 0.006; Group 4 vs. Group 6: *t*(34) = 5.91, *p* < 0.001], suggesting that participants adopted a strategy closer to maximization. In contrast, participants who trained without feedback (Group 5) showed strategy index that did not differ significantly from matching [that is, zero strategy index, *t*(14) = 0.80, *p* = 0.436, BF_10_ = 0.347], suggesting that participants learned by matching the probability distribution of the presented context-target contingencies. Due to the lower number of participants who improved when trained with uncorrelated feedback (Group 6), the strategy index in this group was lower than zero [*t*(17) = −2.78, *p* = 0.013] and significantly lower than Group 5[*t*(31) = −2.13, *p* = 0.041]. These results suggest that trial-by-trial informative feedback facilitates maximization and learning of temporal statistics.

### Correlating strategies to learning performance

**FIGURE 6:**
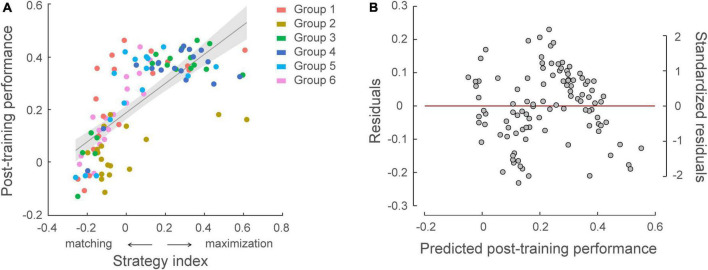
Relating individual decision strategy to learning performance across participants in all groups (*N* = 105). **(A)** Significant correlations of individual decision strategy and post-training performance, that is normalized performance index after training [*r* = 0.717, CI = (0.62, 0.80)]. A skipped Pearson correlation analysis using the Robust correlation toolbox (Pernet et al., 2012) replicated this significant positive correlation following exclusion of five bivariate outliers [*r* = 0.790, CI = (0.72, 0.85)]. Strategy-index values close to zero indicate a strategy closer to matching, while higher positive values indicate a strategy closer to maximization. The color of the dots indicates participant group. **(B)** Residual plot of multiple regression analysis with group membership adjusted. Residuals were plotted against the post-training performance predicted from the multiple regression model, validating the assumptions of linearity and homoscedasticity.

We further tested whether individual strategies relate to learning performance (Figure 6). Combining data across experiments (*N* = 105), there was a significant correlation between participants’ strategy index and post-training performance (i.e., normalized PI after training) (*r* = 0.717, *p* < 0.001), indicating that participants who adopt a strategy closer to maximization show better learning performance. Despite the fact that learning performance varied across groups due to the experimental manipulation (a linear regression model showed group as a significant predictor on post-training performance, *R*^2^ = 0.299, *F* = 8.43, *p* < 0.001), including strategy index as an additional regressor significantly explained 34.7% more variance in post-training performance (*p* < 0.001, Δ*R*^2^ = 0.347). At first glance, this result may appear surprising, as exact matching is expected to result to 100% performance. Interrogating the response distribution across participants showed that most learners with high PI adopted a strategy toward maximization (i.e., responding more than 80% to high probability contingencies) over training. This result is in line with previous findings suggesting that probability maximization is favored when learning complex probabilistic tasks (Lagnado et al., 2006; Wang et al., 2017a).

### Correlating cognitive abilities to learning temporal statistics

Finally, we asked whether cognitive control abilities (i.e., attention, working memory) relate to learning performance. Selective attention and working memory were assessed before training on the prediction task using the Useful Field of View task and visual short-term memory tasks, respectively. We observed individual variability in cognitive tasks across participants. Performance in selective attention–as measured by SOA duration needed for separating targets from cluttered distractors–ranged from 16 to 165 ms, and performance in working memory–as measured by number of items which were correctly memorized–ranged from 3.4 to 9.5 number of items. There was a significant correlation between selective attention and working memory scores (*r* = −0.286, *p* = 0.003) across participants in all groups. Multiple regression analyses showed that selective attention and working memory: (a) explained significantly [*F*(2,102) = 7.04, *p* = 0.001] 12.1% of the variance in strategy index (*R* = 0.348) in the prediction task across all participants (*N* = 105), (b) explained significantly [*F*(2,102) = 6.46, *p* = 0.002] 11.2% of the variance in post-training performance (*R* = 0.335).

**FIGURE 7:**
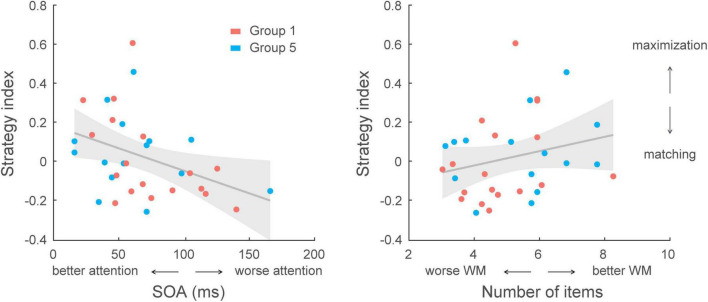
Correlating cognitive skills with decision strategy across participants in Group 1 and Group 5 (*N* = 33). **Left**, correlation of selective attention scores with strategy index. A lower score (SOA: stimulus onset asynchrony; i.e., shorter display duration) indicates better performance in the selective attention task that relates to decision strategy closer to maximization [*r* = –0.394, CI = (–0.59, –0.20)]. **Right**, correlation of working memory (WM) scores with strategy index. A higher score (larger number of items in the display) indicates better performance in the working memory task. However, no significant correlation was found between working memory capacity and decision strategy [*r* = 0.246, CI = (0.01, 0.49)].

Given that strategy varied across groups due to experimental manipulation, a further multiple regression analysis accounting for group effect showed that group and cognitive abilities (selective attention, working memory) explained significantly [*F*(7,97) = 5.30, *p* < 0.001] 27.7 % of the variance in strategy index (*R* = 0.526), and group had the strongest impact on decision strategy (*p* < 0.001, *R*^2^ = 0.234). Interestingly, we found that selective attention rather than working memory was better at predicting the strategy adopted during training (Supplementary Table 1); that is, excluding the variation accounted for by group (i.e., experimental manipulation), a model with selective attention as an additional regressor significantly explained 3.3% more of the variance in strategy index (*p* = 0.038). However, we did not observe a significant impact of working memory in the model (*p* = 0.104, BF_10_ = 1.102). Similar results were found when conducting correlations with combined data from Group 1 and Group 5 (no group difference in strategy index, *t*(31) = −0.44, *p* = 0.660, BF_10_ = 0.360). There was a significant correlation between selective attention and strategy index (*r* = −0.394, *p* = 0.023), but no significant correlation between working memory and strategy index (*r* = 0.246, *p* = 0.168, BF_10_ = 0.538) (Figure 7). These results suggest that selective attention is a key predictor of decision strategy; that is, it is likely that selecting the most probable outcomes when maximizing facilitates learning of temporal statistics.

## Discussion

Extracting the statistics governing event streams is critical for adaptive behavior in rapidly changing environments. Here we employed a novel behavioral paradigm to test participants’ predictions following multiple days of exposure to probabilistic structures. In contrast to most previous studies which focus on implicit measures of sequence learning such as familiarity judgments or reaction times, our approach allows us to track individuals’ responses over time and interrogate the decision strategy adopted during training. This approach allows us to develop a direct and reliable measure to assess group-level performance and individual differences in structure learning (Siegelman et al., 2017). Our findings demonstrate that exposure to temporal sequences facilitates our ability to extract their structure and predict upcoming events; an improvement that lasts for a prolonged period following training (up to 4 weeks). We show that this learning of predictive structures is maintained under uncertainty that relates to the characteristics of the temporal sequences and task feedback. In particular, learners adapt their behavior to changes in the sequence design, rate of stimulus presentation and feedback. Further, attentional skills account for variability in decision strategy that significantly relates to individual structure learning ability. Our findings advance our understanding of structure learning in four main respects.

First, we show that less discriminable contingency probabilities compromised learning performance. This is consistent with previous work (Thiessen et al., 2013; Hasson, 2017; Okano et al., 2021), showing that probability of stimulus occurrence is key for extracting spatiotemporal structures. For example, previous studies have shown that sequences of syllables with high conditional probabilities are perceived to correspond to words, while syllable transitions with low predictability are more likely to be perceived as word-boundaries (Saffran et al., 1996b). Our results showed that participants failed to extract the underlying first order Markov structure (i.e., identify the correct context-target contingencies) in sequences with highly similar contingency probabilities. To further quantify performance, we applied a response-tracking approach (Wang et al., 2017a) that monitors participant predictions across trials and extracts dynamic changes in relation to the rule used to generate the sequences–that is, context length (e.g., for level-1 Markov model, context length = 1, that refers to immediately preceding item for identifying the sequence structure), and the context-target contingencies between stimuli. This analysis showed that decreasing the difference in occurrence probability resulted in impaired performance, which is not only due to poor estimation on the conditional probabilities, but indicates impaired ability to extract the correct context length (behaviorally relevant rules) used to generate the sequences (Supplementary Figure 1). These results reveal that probability of context-target contingencies plays a key role in learning temporal structure.

Second, we asked whether temporal uncertainty influences learning of predictive structure. Previous studies have shown that temporal variability and uncertainty disrupts temporal expectation and impairs performance (Nobre et al., 2007). Presenting stimuli at a regular rhythm or at the expected time has been shown to facilitate action preparation and execution (e.g., reduced reaction times and saccade latencies) (Niemi and Näätänen, 1981) and enhance perceptual judgments (Lasley and Cohn, 1981; Westheimer and Ley, 1996; Rolke and Hofmann, 2007; Rohenkohl et al., 2012). In contrast to this previous work, we found that learning of predictive structures was maintained when disrupting the rhythmic presentation of the sequence by introducing temporal jitter. Interestingly, learners presented with temporally jittered sequences adopted a strategy closer to maximization, suggesting that maximizing may facilitate learning of temporal structure under temporal uncertainty. It is likely that our participants focused on the probabilistic associations between symbols rather than the sequence rhythm, as our prediction task requires the participants to make an explicit judgment about the expected stimulus. This is consistent with previous work suggesting that humans are rational probabilistic learners and able to extract organized structures from ambiguous information [e.g., feature correlations in multidimensional sequences (Turk-Browne et al., 2008)] in a flexible manner (Aslin and Newport, 2012). Further, temporal jitter may result in increased cognitive load. Our results suggest that adopting a strategy closer to maximization facilitates structure learning under conditions of higher task demands, consistent with previous work showing that participants adopt a strategy closer to maximization when learning more complex probabilistic sequences and tasks after training (Lagnado et al., 2006; Wang et al., 2017a).

Previous behavioral and neurophysiological studies have suggested that temporal uncertainty induced by varying the regularity of rhythmic stimulus streams influences stimulus processing at a perceptual level (Rolke and Hofmann, 2007; Schroeder et al., 2010; Rohenkohl et al., 2012). Entraining the brain to rhythmic events has been shown to facilitate sensory processing (Lakatos et al., 2008; Schroeder and Lakatos, 2009; Nobre and van Ede, 2018). Following exposure to structured sequences increased neural entrainment to embedded patterns was observed over a range of brain regions from sensory to frontal areas. This in turn may trigger a top-down modulation, reflecting the successful perceptual grouping of individual stimuli into cohesive units (e.g., syllables into words) (Batterink and Paller, 2017; Park et al., 2020; Moser et al., 2021). Our results suggest that despite temporal interference at stimulus level, entrainment to larger embedded patterns may still be retained in brain circuits beyond sensory processing. In particular, we have previously shown that distinct brain circuits relate to individual strategies for learning temporal statistics (Wang et al., 2017b; Karlaftis et al., 2019): probability matching engages occipitotemporal and ventral caudate regions, whereas maximization engages fronto-striatal circuits (i.e., dorsolateral prefrontal cortex, cingulate, sensory–motor regions, and putamen). Irregular stimulus presentation may disrupt perceptual processing in visual cortico-striatal circuits that have been shown to relate to probability matching for structure learning. Therefore, it is possible that structure learning under temporal uncertainty recruits fronto-striatal circuits that support learning by maximization rather than matching facilitating learners to flexibly adapt their decision strategy and learn the environments statistics.

Third, we test whether feedback modulates decision strategy and learning of predictive statistics. Feedback is known to play a key role in learning new skills from simple feature processing to complex social interactions (Kluger and DeNisi, 1996). Theoretical work has proposed that supervised, error-correcting learning mechanisms rely on external feedback (Dayan and Abbott, 2001; Petrov et al., 2005; Liu et al., 2014). Yet, previous work on statistical learning has shown that learning of spatiotemporal regularities may occur implicitly (i.e., by mere exposure rather than external feedback) (Perruchet and Pacton, 2006). Our results demonstrate that participants were able to extract the underlying sequence structure without any feedback; that is, participant who received no feedback or sparse performance feedback (i.e., mean performance feedback quantitatively across a block of sixty trials) performed similarly in the prediction task. However, trial-by-trial correct feedback enhanced task performance and resulted in learners adopting a strategy closer to maximization than matching. In contrast, random feedback that was uncorrelated to the participants responses compromised learning substantially. Our results are consistent with previous work showing that informative feedback shifts decision strategy toward maximization in probabilistic choice tasks (Shanks et al., 2002; Lagnado et al., 2006). That is, trial-by-trial feedback supports error correction, reducing uncertainty and facilitating a decision strategy that delivers best outcomes and increased reward. In particular, trial-by-trial feedback may encourage a maximization strategy, resulting in higher probability of selecting the correct response (e.g., 80%, in contrast to matching strategy, of 68% hit rate), consistent with reinforcement learning and rational choice theory (Vulkan, 2000; Shanks et al., 2002; Erev and Barron, 2005; Newell et al., 2013).

Finally, we show that individual strategies and performance in learning predictive statistics correlate with cognitive capacity as indicated by attentional and working memory skills. The role of attention and working memory in statistical learning remains debated (Conway, 2020). It is possible that working memory is involved in the encoding of multiple sequence items, facilitating learning of temporal statistics; yet the role of working memory in sequence learning remains controversial (Janacsek and Nemeth, 2013). Further, some studies propose that selective attention may gate learning of statistics; that is, regularities are only learned when the stimuli are attended (Turk-Browne et al., 2005; Richter and de Lange, 2019), while others argue that extracting regularities is a consequence of attentional processing (Pacton and Perruchet, 2008). Statistical learning has been proposed to involve a multicomponent learning system that relates to stimulus encoding, retention and abstraction, with each component of this system depending on attention or working memory to a different degree (Arciuli, 2017). Although attention and working memory have traditionally been considered to be distinct cognitive processes, recent studies propose an overlap between the brain systems (e.g., frontal circuits) that support these processes (Awh et al., 2006). Previous work has demonstrated a competitive interaction between higher cognitive functions and implicit statistical learning (Thompson-Schill et al., 2009; Ambrus et al., 2020). For example, depleting cognitive control system enhances adults’ implicit but not explicit word segmentation abilities (Smalle et al., 2022). However, in our experimental paradigm prolonged exposure to visual statistics in combination with prediction judgments may enable participants to evoke explicit attention-based processes and search for efficient learning strategies. Here we provide evidence that participants with better attentional and working memory skills adopt a strategy closer to maximization and show improved structure learning. Our results are consistent with previous work showing a positive correlation of attentional and working memory abilities with learning performance in a prediction task in both mild cognitive impairment patients and healthy controls (Baker et al., 2015). These cognitive skills are thought to: (a) implicate a frontoparietal network (Corbetta and Shulman, 2002; Corbetta et al., 2008) involving strategy updating for future predictions (Danckert et al., 2012), (b) be coded by a multiple-demand system for intelligent behavior such as sequential mental programming with changing context (Duncan, 2010). A recent study showed that contextual associations were learned only when participants were explicitly instructed to attend to the regularities and provided with corrective feedback. This task-relevant contextual sensory prediction was accompanied by progressively higher expectation suppression across the cortical hierarchy, from executive control regions downstream to task-relevant sensory areas (Ferrari et al., 2022). Interestingly, our results demonstrate that selective attention is a stronger predictor of maximization strategy than working memory. This is likely due to the fact that extracting probabilistic conjunctions is more relevant in the context of our prediction task than memorizing sequences. Consistent with previous computational work proposing a key role of attentional selection in learning (Dayan et al., 2000; Yu and Dayan, 2005), our findings suggest that selective attention may facilitate the selection of the most probable outcomes (i.e., adopting a maximization strategy), reducing uncertainty and supporting learning of temporal statistics.

In sum, our findings provide evidence for flexible learning of predictive statistics; that is, individuals adapt their decision strategy to learn the underlying structure of events in the face of sensory uncertainty and predict upcoming events. Adopting a maximization strategy facilitates learning by reducing sensory uncertainty. Further corrective feedback enhances maximization, reducing uncertainty and supporting our ability to learn the structure of the environment and make successful predictions.

Note that quantifying the decision strategies is key for understanding how participants learn the underlying sequence structure. A possible limitation of our approach is that, when probability estimations are very close to each other (e.g., 60% vs. 40%), then discerning strategies (e.g., poor maximizing vs. matching performance) becomes challenging. To this end, using a design with more context–target contingencies of different probabilities and examining how participants respond to low-probability contingencies could be helpful. For example, considering a design with ground truth of 70%, 20%, 10%, exact matching would result in response probabilities: 70%, 20%, 10% while poor maximization would result in response probabilities: 70%, 15%, 15%. In addition, future work may investigate the brain plasticity mechanisms that mediate our ability for this flexible structure learning under uncertainty.

## Data availability statement

The raw data supporting the conclusions of this article will be made available by the authors, without undue reservation.

## Ethics statement

The studies involving human participants were reviewed and approved by the University of Cambridge Ethics Committee, the institutional review board of the Institute of Psychology, Chinese Academy of Sciences. The patients/participants provided their written informed consent to participate in this study.

## Author contributions

RW and ZK designed the research. RW and VG performed the research and analyzed the data. RW, YS, and PT contributed analytical tools. RW, YS, PT, and ZK wrote the manuscript. All authors contributed to the article and approved the submitted version.
