# Supplementary material for: Flexible structure learning under uncertainty
Source: Front Neurosci. 2023 Aug 3;17:1195388. doi: 10.3389/fnins.2023.1195388 (PMC10437075; doi:10.3389/fnins.2023.1195388)
Supplement: Supplementary file 1 [file Image_1.pdf]

Supplementary figure

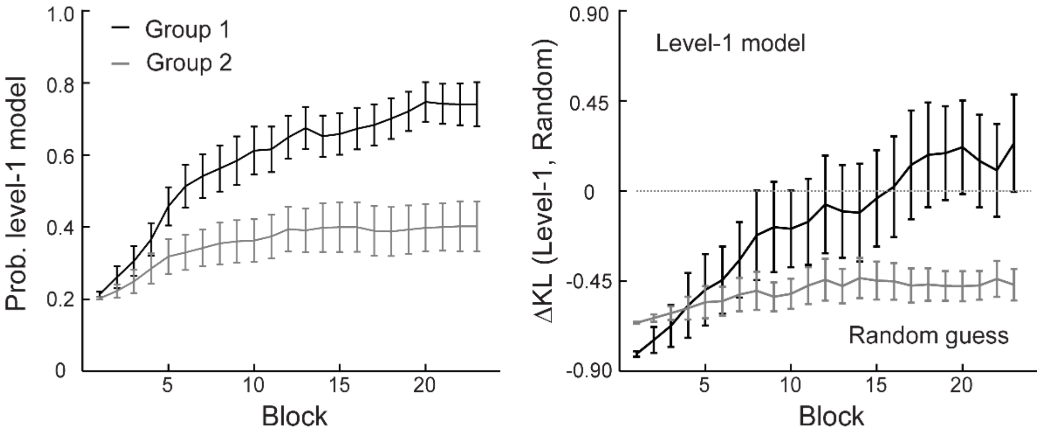

Supplementary figure. Response tracking analysis. We dynamically tracked the participants' predictions across trials using a weighted combination of multiple Markov processes (i.e., zero-, first-, second-order), which allows us to capture changes in performance that relates to extracting the context length that governs the sequences, and extracting context-target contingencies to generate a prediction about the next stimulus given the current context (Wang et al., 2017a). Left shows the context-length learning indicated by the evolution of the coefficients of the individual mixture components (i.e. level-1) across training blocks. Right shows learning predictive contingencies indicated by ΔKL-curves based on the comparison of underlying Markov model and a baseline model (i.e. random guess). ΔKL values above zero indicate that the participant responses approximated the Markov model that generated the sequences. Error bars indicate SEM. Data are shown for Group 1 (black lines) and Group 2 (gray lines).
