# Supplementary material for: Flexible structure learning under uncertainty
Source: Front Neurosci. 2023 Aug 3;17:1195388. doi: 10.3389/fnins.2023.1195388 (PMC10437075; doi:10.3389/fnins.2023.1195388)
Supplement: Supplementary file 2 [file Table_1.pdf]

Supplementary table. The multiple regression analysis: individual decision strategy relating to the experimental manipulation (i.e. Group difference) and the performance in cognitive tasks.

DV: strategy index

**Model**	**Predictor**	**Estimated β**	**Standard Error**	**t**	**p**	**R**	R^2	**Adj.**R^2	ΔR^2	ΔF
M_0	(Intercept)	0.006	0.048	0.129	0.898	0.484	23.4%	19.5%	23.4%	6.045***
	Group2 (1)	-0.028	0.068	-0.415	0.679					
	Group3 (1)	0.163	0.068	2.387	0.019					
	Group4 (1)	0.228	0.068	3.345	0.001					
	Group5 (1)	0.033	0.071	0.462	0.645					
	Group6 (1)	-0.080	0.068	-1.180	0.241					
M_1	(Intercept)	0.103	0.066	1.561	0.122	0.517	26.7%	22.2%	3.3%	4.405*
	ATT	-1.334	0.636	-2.099	0.038					
	Group2 (1)	-0.015	0.067	-0.224	0.824					
	Group3 (1)	0.149	0.067	2.216	0.029					
	Group4 (1)	0.194	0.069	2.807	0.006					
	Group5 (1)	0.020	0.071	0.277	0.782					
	Group6 (1)	-0.074	0.067	-1.099	0.274					
M_2	(Intercept)	-0.133	0.098	-1.368	0.174	0.504	25.4%	20.9%	2.1%	2.696
	WM	0.027	0.017	1.642	0.104					
	Group2 (1)	-0.045	0.068	-0.664	0.509					
	Group3 (1)	0.151	0.068	2.220	0.029					
	Group4 (1)	0.196	0.070	2.796	0.006					
	Group5 (1)	0.022	0.071	0.308	0.759					
	Group6 (1)	-0.096	0.068	-1.410	0.162					
M_3	(Intercept)	-0.011	0.120	-0.095	0.924	0.526	27.7%	22.5%	4.3%	2.872^{+}
	Group2 (1)	-0.029	0.068	-0.429	0.669					
	Group3 (1)	0.143	0.067	2.115	0.037					
	Group4 (1)	0.176	0.071	2.496	0.014					
	Group5 (1)	0.014	0.071	0.192	0.848					
	Group6 (1)	-0.086	0.068	-1.269	0.207					
	WM	0.020	0.017	1.151	0.253					
	ATT	-1.137	0.657	-1.730	0.087					

ATT denotes the selective attention; WM denotes the working memory.
M_0 includes Group as factor to predict strategy index, M_1 and M_2 include one dimension of cognitive abilities (attention or working memory, respectively) as an additional factor, and M_3 includes both attention and working memory as additional regressors.
Mentioned Δ values in Model 1-3 refer to the comparisons between the underlying model and M_0.
*** $p < 0.001$, * $p < 0.05$, $^{+}$ denotes marginal significance ($p = 0.061$).
